# Ionophore Toxicity in Animals: A Review of Clinical and Molecular Aspects

**DOI:** 10.3390/ijms24021696

**Published:** 2023-01-15

**Authors:** İlksen Berfin Ekinci, Agnieszka Chłodowska, Małgorzata Olejnik

**Affiliations:** Department of Basic and Preclinical Sciences, Institute of Veterinary Medicine, Faculty of Biological and Veterinary Sciences, Nicolaus Copernicus University in Toruń, 11 Gagarina St., 87-100 Torun, Poland

**Keywords:** ionophores, ionophore toxicity, poultry

## Abstract

For many years, ionophores have been used to control coccidiosis in poultry. However, misuse of ionophores can cause toxicity with significant clinical symptoms. The most critical factors influencing ionophores’ toxicity are administration dose, species, and animal age. Although clinical signs of ionophore intoxication are well studied, the toxicity mechanisms of the ionophores at the molecular level still are not fully elucidated. This review summarizes the studies focused on polyether ionophores toxicity mechanisms in animals at the clinical and molecular levels. Studies show that ionophore toxicity mainly affects myocardial and skeletal muscle cells. The molecular mechanism of the toxication could be explained by the inhibition of oxidative phosphorylation via dysregulation of ion concentration. Tiamulin-ionophore interaction and the synergetic effect of tiamulin in ionophore biotransformation are discussed. Furthermore, in recent years ionophores were candidates for reprofiling as antibacterial and anti-cancer drugs. Identifying ionophores’ toxicity mechanisms at the cellular level will likely help develop novel therapies in veterinary and human medicine.

## 1. Ionophores

### 1.1. Ionophore Structure and Mechanism of Action

Ionophores are lipid-soluble molecules that transport specific cations through biological membranes. The lipophilic feature of ionophores allows cations to cross the cellular and subcellular membranes [1]. All ionophores share a typical chemical structure. The inner part of the ionophore is a hydrophilic feature where cations bind, whereas the outer part is a hydrophobic hydrocarbon structure that allows the ionophore to pass the phospholipid bilayer easily.

Ionophores are classified into two groups: neutral ionophores and carboxylic ionophores. Neutral ionophores generate charged complexes with cations and are highly toxic. Carboxylic ionophores form complexes with the cations. Neutral ionophores disturb the membranes, and carboxylic ionophores promote the exchange diffusion of electrically neutral cations [2]. The polyether carboxylic ionophores always contain one carboxylic group, tetrahydropyran and tetrahydrofuran rings, and several hydroxyl and ketone groups [3]. Polyether carboxylic ionophores can be divided into three subgroups: monovalent polyether ionophores (salinomycin, monensin, and narasin), monovalent glycoside polyether ionophores (maduramicin and semduramicin), and divalent polyether ionophores (lasalocid) [4].

The polyether ionophores contain oxygen atoms that allow making a pseudo-cyclic cage with cations and/ or hydrogen atoms and they carry the cations in three mechanisms that are electroneutral (biological), electrogenic, and biomimetic transport [3,5]. The chemical structure of the ionophores and the cell environment determine the transport mechanisms [6]. In the electroneutral transport, polyether ionophore releases a proton (H^+^) from its carboxyl group to the outer part of the biological membrane, and the metal cation binds to the negatively charged ionophore. The cation uploaded ionophore releases the cation to the inner part of the membrane, gains a proton, and returns to the stable form [7]. In the electroneutral transport mechanism, the cation or hydrogen binds to the ionophore and neutral salt occurs, the polyether ionophore replaces itself as an acidic form respectively, and only uncharged cation molecules are transported through the lipid membrane. This transportation occurs only in cells with an alkaline environment, and this causes the deprotonation of the polyether ionophore.

**Figure 1:**
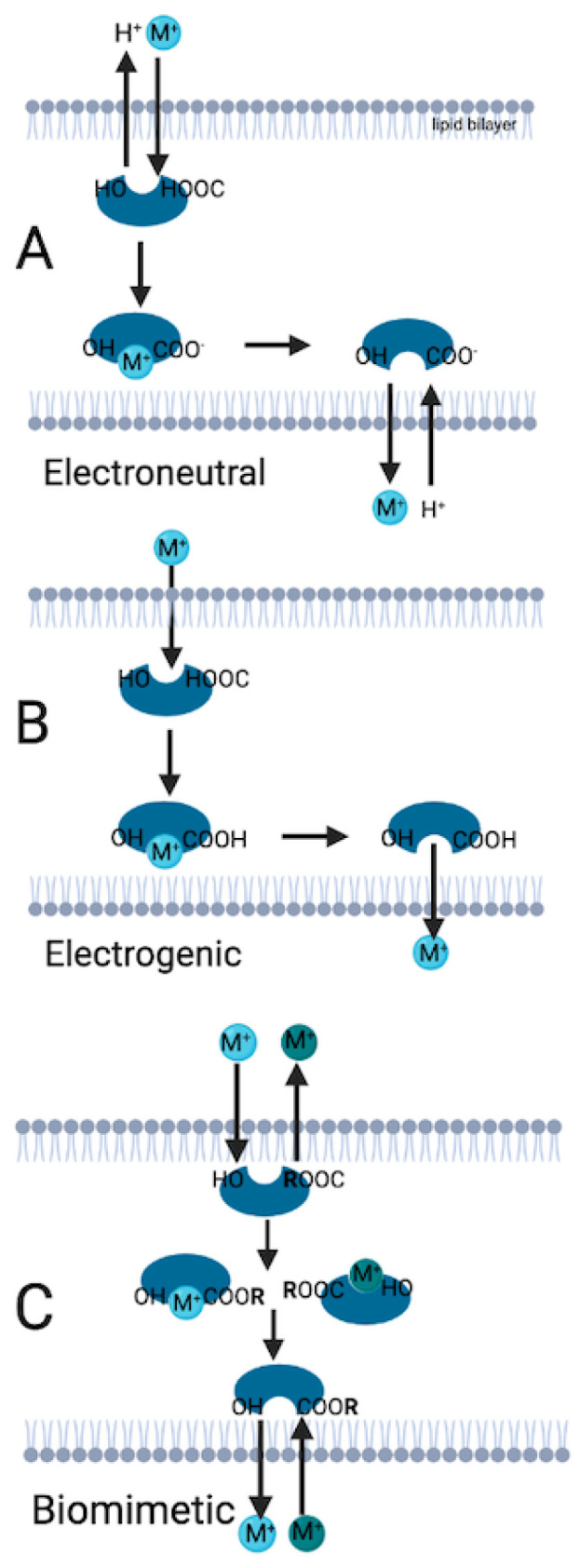
Ionophore transport mechanisms. (**A**): Electroneutral transport occurs when the ionophore is charged negatively because of the proton released from the carboxyl group. (**B**): In the electrogenic transport, the ionophore is uncharged, and transport occurs throughout the lipid bilayer membranes. (**C**): If the ionophore has an R group, biomimetic transport occurs. M: Representative illustration of cation molecule. R: Radical group. (Illustration is created in Biorender.com accessed on 26 June 2022).

Electrogenic and biomimetic mechanisms are alike because they both can operate without the basic/alkaline microenvironment of the cell and allow molecule transportation without deprotonation of the polyether ionophore [3]. In electrogenic transport, an uncharged ionophore transports the cation from the outer to the inner part of the lipid bilayer [8,9]. Biomimetic transport is basically mimicking living organisms’ biological and physiological features [10,11]. The ionophore contains a radical group ® such as ester and amide, which exchanges two metal cations. One cation moves to the inner part of the lipid bilayer membrane, whereas the other cation moves to the outer part of the lipid membrane simultaneously [3]. The design and usage of biomimetic transporters and transportation could allow the transport of selective ion molecules faster, more efficiently, and with less energy [12]. Using biomimetic transport, modified polyether ionophores can produce more efficient results against diseases caused by ion dysregulation, such as coccidiosis in animals. Transport mechanisms of cation-uploaded ionophores are summarized in Figure 1.

### 1.2. Ionophores in Coccidiosis

Ionophores are commonly used as parasiticides to prevent animal coccidiosis [8]. Most ionophores are produced by Gram-positive bacteria, mainly the *Streptomyces* genus [9]. Over 50 microorganisms produce carboxyl ionophores. To date, over 120 polyether carboxylic ionophore antibiotics have been identified. However, only six have been approved and are used as anticoccidials for livestock. These are monensin, lasalocid, salinomycin, narasin, maduramicin, and semduramicin (Table 1) [10,11,12].

Coccidiosis is a protozoa invasion occurring in nearly every vertebrate animal. There are thousands of described host-specific coccidian species [13]. The disease occurs under high moisture and temperature husbandry conditions. In poultry, coccidiosis is caused by *Eimeria* spp. [14] and is estimated to cost around GBP 10.4 billion globally at 2016 prices, equivalent to GBP 0.16/chicken produced [15]. Coccidiosis manifests in severe diarrhea, poor weight gain, and sometimes mortality. The mucus membrane in the small intestine shows symptoms of inflammation. The feces of infected animals transmit coccidia [13].

The mechanism of antiparasitic action of ionophores is as described above; they disrupt membrane potential because of the changing ion gradient in protozoa [16]. For instance, lasalocid increases intracellular Na^+^ and Ca^+2^ concentration in protozoa [10]. These ion imbalances cause osmotic pressure and metabolic dysregulation. Finally, protozoa start swelling and exploding. Different ionophores are effective against different protozoa species.

According to the European Union Register of Feed Additives, monensin, salinomycin, lasalocid, semduramicin, and narasin are registered as coccidiostats in poultry. All of them may be used in chickens. In turkeys, only monensin, lasalocid, and maduramicin are utilized. Furthermore, lasalocid is used for the treatment and prophylaxis of pheasants, guinea fowl, quails, and partridges.

Producing organisms for ionophores, affected protozoa species, recommended concentration in feed, and withdrawal periods of ionophores are summarized in Table 2.

## 2. Toxicity of Ionophores

Accidental usage or incorrect dosage of the ionophores causes toxicity [1]. Ionophore toxicity is characterized by degeneration of the myocardium, anorexia, poor weight gain, and muscle weakness [9].

Ionophores are responsible for transporting cations across the inner and outer plasma membranes. Therefore, the primary molecular mechanism of ionophore toxicity is an imbalance of the ion gradient in the cells. The ionophoric activity may change the normal ion concentration gradient, and this causes cellular ion imbalance, pH change, and disruption of the plasma membrane [6,33]. Due to their chemical structure, ionophores cause an accumulation of Ca^2+^ ions in cells, and also, they can promote lipid peroxidation. At the cellular level, ionophore toxicity affects mitochondria and plasma membranes. As a result of the ion concentration imbalance, mitochondria-mediated oxidative phosphorylation is inhibited. Cells of the myocardium and skeletal muscles are affected the most, probably because of their high metabolic activity [1,3,4,34].

Although the mechanism of ionophore toxicity at the cellular level is common for all animals, the clinical manifestation differs greatly depending on animal species (Table 3). It is important to remember that the median lethal doses (LD_50_) reported here represent acute exposure, differing greatly from field conditions. The differences in species susceptibility, together with attempts to explain these differences and the toxicity of ionophores after (sub)chronic exposure, will be discussed in further paragraphs regarding the toxicity of respective ionophores.

Some biomarkers have been used for the determination of ionophore toxicity. For instance, some studies found that numerous serum proteins were increased, such as creatine kinase (CK), aspartate aminotransferase (AST), lactate dehydrogenase (LDH), and alkaline phosphatase (ALP) [2]. The clinical importance of those biomarkers will be discussed in later paragraphs.

### 2.1. Salinomycin Toxicity

The molecular mechanism of toxicity is the same as for pharmacological action. Salinomycin impairs mainly the K^+^ efflux through the cellular membranes. Disruption of ion balance and membrane potential leads to pH alteration in cells. K^+^ efflux into the cytoplasm decreases the pH. Reduction of pH increases intracellular Ca^2+^ levels and injures mitochondria via mitochondria membrane interruption and production of reactive oxygen species (ROS) [39].

In broiler chickens, the salinomycin non-toxic dose was determined to be 60 ppm [39]. Compared to chickens, turkeys are more sensitive to the intoxication of salinomycin [40]. Between 44 and 60 ppm of salinomycin in feed have been found toxic in turkeys. Older turkeys were more susceptible to salinomycin intoxication [41,42,43,44]. Griffiths et al. investigated the fall of 400 point-of-lay turkeys, which occurred seven days after commencing a new batch of feed containing 40–47 ppm of salinomycin. Two feeding trials were undertaken, in which feed was supplemented with either 9 or 50 ppm salinomycin. They found the 50 ppm diet toxic but also demonstrated that ingestion of salinomycin at relatively low concentrations (9 ppm) may negatively affect the health and productivity of laying turkeys [45].

The field data do not always correspond with established toxicity levels. Halvarson et al. showed 34% mortality correlating with only 24 ppm of salinomycin in the feed [44]. There are several documented cases of accidental use of broiler concentrate in turkeys, leading to high mortality in a flock [43,46]. A report from Choon Yong showed that 16 to 64 ppm salinomycin doses were highly toxic in 12-week-old turkeys [47]. A study performed by Koutoulis et al. demonstrated that salinomycin used at 29.8–94.4 ppm in feed caused severe toxicity in 20-to-35-day-old turkeys [43]. Furthermore, adult turkeys showed clinical signs of intoxication after 27–56 ppm salinomycin administration [44]. The 30-week-old turkeys showed similar toxicity pathology after 50 ppm salinomycin treatment [45].

Although the recommended dosage of salinomycin has been known and well-studied in chickens, there are fewer studies to examine salinomycin toxic dose in other animal species. As different animals can be exposed to salinomycin through cross-contaminated feed, it is essential to determine the non-toxic doses of ionophores. After the accidental poisoning of rabbits with feed containing 26.9 ppm of salinomycin, Peixoto et al. conducted an experiment and established that a concentration above 50 ppm of salinomycin in feed is not lethal but can cause adverse effects on performance, such as growth depression and decrease in food digestion [48]. Al-Wabel et al. performed a study in camels and found that a 15 mg/kg dose of salinomycin was severely toxic [49]. In 1995, a case of acute salinomycin toxicosis in swine was reported. Although the owner fed cows and pigs the same ionophore-contaminated feed, none of the cattle became ill. Pigs died in less than 24 h after the introduction of the contaminated feed, which contained 441–720 ppm of salinomycin [50].

In some cases, even a relatively small dose of salinomycin shows a highly toxic effect. A study performed by Hosseini et al. showed that 0.5 mg/kg salinomycin alters biochemical parameters and affects the myocardial tissues of sheep [51].

Studies investigating the toxic effects of salinomycin are focused mainly on in vivo experiments. They include the administration dose of the ionophore, the observations, and the examination of the clinical symptoms of the animals. However, in vitro approaches can allow use of high-throughput approaches and analyses to identify toxicity mechanisms in animals at the cellular level.

Using in vitro experiments, Gao et al. found that salinomycin causes myocardial hyperemia, myocardial fiber degeneration, and mitochondria damage and induces osmotic pressure in primary chicken myocardial cells isolated from chicken embryos. Cell viability was also decreased in chicken myocardial cells in a concentration-dependent manner [52]. Similar to this study, Cybulski et al. showed that salinomycin reduced cell viability in a chicken hepatoma cell line in a dose-dependent manner [53]. These in vitro studies determined that salinomycin has an adverse effect on the heart and liver; the increased toxic dose of salinomycin causes a severe influence on these organs. In both myocardial and hepatic cells, salinomycin follows a similar molecular signaling pathway, resulting in the disintegration of the cell membrane [52,53].

The toxicity symptoms of salinomycin could impact the whole body and adversely affect the lives of animals. According to Diaz and colleagues, the spleen and pancreas were also affected in broiler chickens after salinomycin administration in a time- and dose-dependent manner [54]. In nearly every case, atrophy of the spleen is observed, as one of the gross lesions, although none of the symptoms or lesions are pathognomonic [43,44,46]

A study performed by Rajaian et al. investigated the effect of various oral doses of salinomycin in ruminants [55]. After salinomycin administration, the following pathophysiological symptoms occurred: anorexia, tachycardia, heart attack, muscle weakness, and paralysis [55]. Similar to this study, Ashrafihelan et al. found the same symptoms in sheep after salinomycin treatment [56]. A toxic dose of salinomycin could cause anorexia, muscle weakness, and myocardial, liver, and kidney damage in camels [49]. Rizvi et al. concluded that the recommended dose of salinomycin, which is 60 ppm, did not influence the liver and kidneys, whereas the 120 ppm and 180 ppm doses had a statistically significant adverse impact on the liver and kidney samples of chickens [57]. Salinomycin toxicity could cause hypertrophy of the organs. Spleen and pancreas weights were increased relatively after salinomycin treatment in chickens [54].

Although the molecular mechanism of salinomycin has not been fully understood yet, some biochemical parameters and histopathology analysis could help clarify the possible toxicity mechanisms of ionophores. For instance, it was demonstrated that 60 mg/kg and 120 mg/kg doses of salinomycin increased AST and ALT levels in broiler chickens’ serum [58]. These biomarkers indicate liver damage. Furthermore, total cholesterol, triglyceride, low-density lipoprotein (LDL), bilirubin, urea, and creatine were found to be increased. This study may prove that salinomycin toxicity affects multiple tissues in chickens. Hosseini et al. found that AST, ALT, CK, and LDH were significantly increased after administration of the toxic doses of salinomycin in sheep compared to the untreated samples [51].

Using biochemical biomarkers is a helpful tool in detecting toxicity levels in organs. However, identifying salinomycin toxicity at the cellular level is a great challenge. The intoxication mechanism may cause free radicals production by disturbing antioxidant defenses in chickens [59].

In ruminants, salinomycin significantly decreased serum glucose levels and affected energy metabolism [55]. In addition, because the ion gradient was imbalanced, cellular ATP concentration changed. For this reason, glucose depletion occurred in the blood, and cellular intake of glucose increased. In addition, increased intracellular Ca^2+^ levels might also increase blood glucose concentration in ruminants.

Studies lack consistency on whether salinomycin affects egg production. There are noted cases of poultry (both chickens and turkeys) being fed toxic doses of salinomycin and developing toxicosis symptoms, but with no adverse effect on production variables reported [18,44,60]. Other studies suggest that toxic doses of salinomycin may induce a drop in egg production, which corresponds to a higher mortality rate [43,44]. Jones et al. showed that salinomycin at 60 ppm, which is still considered a non-toxic dose in broilers, when fed to the hens reduced hatchability of the eggs [60] and this must be taken into account when investigating salinomycin toxicity.

### 2.2. Monensin Toxicity

Monensin toxicity mainly manifests with reduced feed intake, poor weight gain, decreased growth rate, and anorexia. Monensin could cause energy depletion and protein retention in chickens [61]. Similar to this result, it was found that monensin toxicity correlated with muscle weakness and affected myocardium and skeletal muscle cells [62]. However, Rath et al. found that monensin did not weaken muscles and tendons significantly [63]. These two studies showed that monensin toxicity might vary, depending on the dose of monensin, duration of monensin administration, and age of chickens.

Some studies stated that after monensin exposure, the formation of lipid vesicles and swelling of mitochondria occurred in the muscles of pigs, horses, and cattle [64]. Although the recommended dose of monensin ranges between 100–125 mg/kg in chickens, Keshavarz et al. showed that already 100 ppm of monensin significantly decreased the growth of 4 weeks-old chickens [65]. Thus, the recommended dose could depend on the chicken’s age. Additionally, the study conducted by Jones et al. determined that monensin at 100 ppm in the feed had an adverse effect on the fertility of broiler-breeder hens [60]. A study conducted in 1974 showed that 121 and 242 ppm doses of monensin were toxic [61].

The doses that are non-toxic for chickens are toxic for turkeys, resulting in symptoms of monensin toxicosis and mortality [44]. Interestingly, the dose toxic for adult turkeys may not affect the young ones. Even at monensin doses of 218 to 300 ppm in feed, no symptoms were observed in young birds [66].

As monensin is selective for Na^+^ ions, the intoxication can cause dysregulation of Na^+^-related enzyme activities. Calo et al. found that monensin disrupts Na^+^/K^+^ ATPase and Ca^2+^-ATPase enzyme activities in chickens, and it causes an imbalance of Na^+^ and Ca^+^ ions in the myocardium and skeletal muscle cells [67]. Another study showed that increased intracellular Ca^2+^ concentration induces damage to skeletal muscle cells. Because of the ion imbalance in skeletal muscle cells, creatine kinase leakage occurs, and Ca^2+^-mediated phospholipase-A partially regulates this process. A study performed by Sandercock et al. showed that monensin increased the uptake of Ca^2+^ ions into the cells in a dose-dependent manner, and 100 µM monensin treatment causes loss of total creatine kinase in chicken skeletal muscle cells [62].

It is difficult to confirm monensin intoxication solely on postmortem examination of the birds. In acute toxicosis, no gross lesions may be observed [68]. Thus, breeders and veterinarians must cooperate. A detailed description of the premortem symptoms in the flock, followed by the residues and feed analysis, is essential for proper diagnosis. A study conducted in 1989 by Vanderkop et al. found that all of the birds that died displayed three or more characteristic clinical signs of monensin intoxication. The signs are sternal recumbency, feed refusal, growth depression, dyspnea, cream-colored diarrhea, muscle stiffness, and muscle weakness [69].

### 2.3. Maduramicin Toxicity

In chickens, the approved dose of maduramicin is 5 mg/kg [70], but such a dose can be toxic in chickens. Five ppm and 10 ppm doses of maduramicin were applied to chickens, and they were considered safe during the first seven days of the administration. After 14 days, both five ppm and 10 ppm doses were determined as toxic for chickens [71]. As for other ionophores used in poultry, toxicity occurrence is often time-dependent [54].

Maduramicin toxicity may cause muscle weakness and affect the liver and heart. However, Singh et al. showed that maduramicin did not significantly impact the mean body weight of chickens, whereas other ionophores cause a decrease in the total body weight [71]. Increasing the number of animals in the study or long-term observation of the animals could prove the same results of maduramicin on body weight as the other ionophores. Maduramicin also affects biochemical parameters. Toxic doses of maduramicin decrease erythrocyte concentration and mean corpuscular hemoglobin concentration (MCHC) count, which suggests hypochromic anemia.

Compared to the other ionophores, more studies investigate the maduramicin toxicity mechanism at the cellular level. A study conducted in 2018 has shown that maduramicin induced apoptosis via increasing apoptotic marker mRNA expression, stimulated morphological changes in chickens’ myocardial cells, and decreased cell viability in a dose-dependent manner [72].

Another recent study showed that after maduramicin administration in primary chicken myocardial cells, Ca^2+^ levels were increased. Additionally, maduramicin elevated pro-apoptotic genes’ mRNA expression, whereas it decreased anti-apoptotic genes’ mRNA expression [73].

One of the possible molecular mechanisms of maduramicin toxicity is cellular stress via the formation of ROS. Like the rest of the ionophores, maduramicin causes ion imbalance in cells and could change cellular Ca^2+^ ion concentration. This may cause mitochondrial stress, activation of apoptotic genes post-transcriptional regulation, and mitochondria-related apoptosis.

Using rat myocardial cell lines, Chen et al. showed that maduramicin induced apoptosis via caspase-dependent and independent mechanisms, necrosis, and autophagy in a dose-dependent manner [70]. These in vitro studies showed that the mechanisms of maduramicin toxicity could follow the same molecular patterns in myocardial cells. This pattern may include increased Ca^2+^ concentration, ROS production, and, finally, mitochondria-associated apoptosis activation.

### 2.4. Lasalocid Toxicity

Lasalocid non-toxic doses have been determined to be 75–125 mg/kg in chickens [21,31]. However, this dose may be considered highly toxic for other species. Decloedt et al. determined LD_50_ doses of lasalocid to be 2–3 mg/kg in horses [74]. The mode of action of lasalocid is similar to other ionophores; lasalocid toxicity causes anorexia, lethargy, muscle weakness, and myocardial cell dysregulation [74]. Using chicken hepatoma and rat myoblasts cell lines, Radko et al. carried out a study to determine the lasalocid toxic effect. It was found that lasalocid decreased cell viability and total cellular protein in both cell lines in a dose-dependent manner (1–250 µM), whereas it increased the release of LDH [75]. Like the other ionophores, lasalocid increases levels of cellular proteins and liver-associated enzymes in chicken and rat. It suggests the similarity of molecular mechanisms of lasalocid toxicity.

## 3. Interaction of Tiamulin with Ionophores

Tiamulin is a semi-synthetic antibiotic derived from pleuromutilin. It is used to treat *Serpulina* and *Mycoplasma* spp. infections in poultry. In chickens and turkeys, the recommended tiamulin dose has been determined as 250 mg/kg and 125 mg/kg, respectively [76].

The administration of tiamulin together with ionophores causes muscle damage and cardiomyopathy in broiler chickens [77]. Umemura et al. found that monensin and tiamulin, when administered together, caused myopathy, muscle weakness, anorexia, depression, and drowsiness in chickens [78]. The coadministration of maduramicin and tiamulin decreased the total body weight and caused changes in skeletal muscles and myocardium in broiler chickens [79]. Tiamulin given with semduramicin negatively affected body weight parameters in broiler chickens in the third week; however, at day 35, no adverse effects were observed on the body weight parameters [80].

Weisman et al. determined that tiamulin with monensin caused sickness and mortality in turkeys at the age of 26 days [81]. However, a study by Kantor et al. showed that maduramicin-tiamulin administration boosted weight gain in broilers; maduramicin anticoccidial activity also improved when administered with tiamulin [82]. Furthermore, Vieira et al. showed that the administration of tiamulin (30, 20, and 15 ppm) with salinomycin did not adversely affect broiler performance at 1–21 and 22–42 days. It was also observed that tiamulin improved feed conversion in broiler chickens [83].

These findings show that the effect of tiamulin depends on ionophore type, animal species, and age. On the other hand, the time- and dose-dependent administration of tiamulin with the ionophore could improve treatment effects.

Simultaneous administration of tiamulin and the ionophores could affect the biochemical parameters. A study performed by Sakar et al. found that the administration of monensin and narasin concurrently with tiamulin increased enzymatic levels (CK, ALD, LDH, AST, ALT) in pigs [84]. In addition, monensin with tiamulin increased CK levels almost 10-fold in chickens.

The first findings of tiamulin-ionophore interaction were carried out by Meingassner et al. and showed that tiamulin decreased the metabolic degradation of monensin in chickens [85]. It is assumed that tiamulin could inhibit the oxidative biotransformation mechanism of the ionophores. However, the exact molecular mechanism of interaction between ionophores and tiamulin has not yet been fully understood. To better understand the significance and mechanism of this interaction, it is imperative to comprehend the modes of ionophore metabolism.

Biotransformation of the ionophores is driven by the cytochrome P450 (CYP450) enzyme family [86,87]. However, the importance of CYP450 and specific routes of metabolism will depend on animal species and ionophore type. For instance, monensin biotransformation via CYP450 activity differs among species, according to Nebbia and colleagues [88] They found that after monensin administration, the microsomal metabolism (O-demethylation) level was higher in cattle compared to chickens, horses, pigs, and rats. Interestingly, in chickens, the turnover rate of CYP450 was higher than in other species [88]. It has been known that chickens are more resistant to ionophore toxicity [41]. Therefore, the maximum level of microsomal metabolism of monensin in cattle could be related to the cattle being more susceptible to monensin than chickens when exposed to the same dose of the monensin. Schumacher et al. showed that semduramicin and tiamulin interaction had a milder adverse effect on chickens compared to other ionophore-tiamulin interactions [80]. One of the explanations for this may be that the biotransformation of semduramicin might be regulated by pathways other than the CYP450 mechanism, which is inhibited by tiamulin.

It has been known that tiamulin is responsible for inhibiting CYP450 enzyme activities [89]. A study by Zweers-Zeilmaker et al. found that tiamulin is an effective inhibitor of CYP450 enzymes in cattle and goats [90]. Similar to this research, it was found that tiamulin inhibits monensin O-demethylation in rat hepatic microsomes. This study shows that the inhibition of monensin biotransformation could be related to CYP450 enzyme inhibition via tiamulin [87]. Radko et al. found that salinomycin-tiamulin toxic interaction correlated with the tiamulin concentration and the inhibition of CYP450 enzymes by tiamulin could decrease the biotransformation of salinomycin in primary human hepatocytes. Interestingly, salinomycin toxicity was synergistically induced by tiamulin in the fibroblast cell line Balb/c 3T3 [89]. The synergetic interaction occurring in the non-metabolizing cells shows that different mechanisms in these cell types could play a role in tiamulin-ionophore interaction.

Furthermore, Witkamp et al. demonstrated the synergetic interaction of tiamulin on CYP450 enzymes in rat microsomes [91]. It could be concluded that tiamulin could be a selective inhibitor or inducer of CYP450 enzymes in metabolizing cells as well as in non-metabolizing cells. The tiamulin-CYP450 interaction and tiamulin’s role in this interaction could depend on animal species and the ionophore type. Supporting these findings, Ratz et al. performed a study to understand the molecular mechanism of tiamulin toxicity in chickens and turkeys, and it was found that simultaneous administration of monensin and tiamulin activates CYP450 enzymes in chickens, whereas it did not significantly affect the turkeys [92]. The toxic interaction between monensin and tiamulin could occur due to the induction of CYP450 enzymes via tiamulin administration and the increased formation of metabolites of monensin in chickens. Although this study showed a toxication between monensin and tiamulin in chickens, it could not show evidence of CYP450 inhibition via tiamulin administration. Furthermore, this data showed that tiamulin interaction with the ionophores at the cellular level could depend on animal species. Although most studies suggested that tiamulin inhibits the oxidative biotransformation mechanism of the ionophores via the inhibition of CYP450, the inducing of CYP450 enzymes via tiamulin was observed in some studies.

## 4. Other Clinical Uses of the Ionophores

The polyether ionophores are used for the treatment of protozoa invasions in animals. However, it was shown that ionophores also have antibacterial properties. Mostly, ionophores have activity against Gram-positive bacteria in a broad spectrum. Gram-positive bacteria lack the second cell wall layer called lipopolysaccharide; therefore, ionophores have high ion permeability in Gram-positive bacteria compared to Gram-negative bacteria [93]. Salinomycin had no antimicrobial effect on *Campylobacter jejuni* infections in chickens [58]. Another in-vitro study showed that monensin, lasalocid, and laidlomycin polyether ionophores did not affect *Salmonella* and *Escherichia coli* foodborne pathogens in ruminants [74]. Nevertheless, some ionophores have activity against both Gram-positive and Gram-negative bacteria. One of these ionophores is PTB2, a type of zinc ionophore that showed an effective result both in-vivo and in-vitro [94,95].

In investigating ionophores’ antibacterial effectiveness, in-vitro-based studies have been performed to detect the minimum inhibitory concentration (MIC) of the ionophores, classification of the efficiency by type of bacteria, and their effects on biofilm formation. A study performed by Lanckriet et al. showed that salinomycin, narasin, lasalocid, and maduramicin significantly decreased the formation of necrotic enteritis lesions caused by *Clostridium perfringes* in vitro [96]. Ionophores are investigated as antimicrobial agents not only for chickens but also for ruminants. A study carried out in 2010 showed that using in-vitro experiments, low concentrations of salinomycin, narasin, monensin, and lasalocid had an antibacterial activity for Gram-positive bacteria such as *Streptococcus uberis* in ruminants [97]. A recent study determined that polyether ionophores could also be used against methicillin-resistant *staphylococci* [98]. Stefańska et al. showed that in vitro salinomycin significantly affected methicillin-resistant *Staphylococcus epidermis*, including 70% inhibition of biofilm formation [99]. These studies suggest ionophores to be an effective compound for fighting bacterial infections in animals.

Ionophores’ activity against viral infections has also been studied. Jang et al. found that in vitro salinomycin inhibited influenza virus infection. As salinomycin is responsible for ion transport and causes pH alteration in cells, it blocks the migration of the viral nuclear protein of the influenza virus [100] The in vitro study by Svenningsen et al. showed that 11 natural polyether ionophores decreased cell viability in cell lines induced by the SARS-CoV-2 virus [101].

In recent years, the investigation of ionophores’ effects on human cancers has become a popular subject. Numerous in vitro studies determined that ionophores could show anti-cancer activity [3,102,103,104,105].

## 5. Conclusions

Although ionophores have been used as anticoccidial agents in poultry for a few decades, new cases of ionophore-induced toxicosis are still being reported yearly. Whether it is accidental use in a non-target species or a mixing error in the mill or on the farm, it results in the death and illness of animals and severe financial damage suffered by the owner.

The research data concerning safety levels and toxic doses often do not correlate with the field picture. Not seldom it is due to the inadequate zootechnical parameters, the clinical picture of the flock, the animals’ age, or the duration of the drug administration. The critical point is to determine the safe dose of the ionophores in susceptible animals, such as turkeys. Clarifying the mechanisms of toxicity of the commonly used ionophores, their metabolism pathways, and their interactions with other drugs will not only help to minimize the risk of intoxication, but it may also lead to discovering novel therapeutic approaches in poultry and, hopefully, in other species.

## Figures and Tables

**Table 1:** Ionophores approved as anticoccidials.

Ionophore Name	Approval Year	Trade Name
Monensin	1971	Coban
Lasalocid	1976	Avatec
Salinomycin	1983	Bio-Cox, Sacox
Narasin	1988	Monteban
Maduramicin	1989	Cygro
Semduramicin	1995	Aviax

**Table 2:** Features of the ionophores. The recommended concentrations in feed and withdrawal periods are given according to European Union Register of Feed Additives, Edition 07/2022 [17].

Ionophore	Producing Organism	Affected Protozoa Species	The Recommended Concentration in Feed	Withdrawal Period	Reference
Salinomycin	*Streptomyces albus*	*E. tenella*, *E. necatrix*, *E. acervulina*, *E. maxima*, *E. brunetti*, *E. mivati*	50–70 mg/kg	1 day	[18,19,20]
Monensin	*Streptomyces cinnamonensis*	*E. acervulina*, *E. brunetti*, *E. maxima*, *E. necatrix*, *E. tenella*, *E. mivati*	100–125 mg/kg	1 day	[19,21,22]
Narasin	*Streptomyces aureofaciens*	*E. acervulina*, *E. brunetti*, *E. maxima*, *E. necatrix*, *E. tenella*, *E. mivati*	60–70 mg/kg	0 days	[18,23,24]
Maduramicin	*Actinomadura yumaensis*	*E. acervulina*, *E. brunetti*, *E. maxima*, *E. necatrix*, *E. tenella*, *E. mivati*	Authorization expired in 2021		[18,25,26,27]
Semduramicin	*Actinomadura roseorufa*	*E. acervulina*, *E. maxima*, *E. brunetti*, *E. tenella*	20–25 mg/kg	5 days	[14,28,29,30]
Lasalocid	*Streptomyces lasaliensis*	*E. tenella*, *E. necatrix*, *E. acervulina*, *E. brunetti*, *E. mivati*, *E. maxima*	75–125 mg/kg	3 days	[31,32]

**Table 3:** Toxicity of the ionophores to different animal species, expressed as median lethal doses (LD_50_).

Species	LD_50_ [mg/kg b.w.]				Reference
	Lasalocid	Monensin	Narasin	Salinomycin	
Cattle	50–150	20–80			[2,35]
Chicken	71.5	200–214	67	40–44.3	[2,36]
Goat	-	26.4	-	-	[2]
Horse	21.5	1–3	0.8	0.6	[2,36]
Mice	146	70–96	15.8–36.7	57.4	[2,37,38]
Rabbit	40	41.7	11.9–15.5	-	[2,37,38]
Rat	122	28.6–40.1	18.5–40.8	48	[2,37,38]
Sheep	75–350	12	-	-	[35]
Swine		16.7–50	8.9	-	[2,38]
Trout	-	>1000	-	-	[2]
Turkey	253			0.6	[2,38]

## Data Availability

No new data were created.
